# Network analysis of the transcriptional pattern of young and old cells of *Escherichia coli *during lag phase

**DOI:** 10.1186/1752-0509-3-108

**Published:** 2009-11-16

**Authors:** Carmen Pin, Matthew D Rolfe, Marina Muñoz-Cuevas, Jay CD Hinton, Michael W Peck, Nicholas J Walton, József Baranyi

**Affiliations:** 1 Institute of Food Research, Norwich, NR4 7UA, UK; 2 Department of Microbiology, Moyne Institute of Preventive Medicine, Trinity College, Dublin 2, Ireland

## Abstract

**Background:**

The aging process of bacteria in stationary phase is halted if cells are subcultured and enter lag phase and it is then followed by cellular division. Network science has been applied to analyse the transcriptional response, during lag phase, of bacterial cells starved previously in stationary phase for 1 day (young cells) and 16 days (old cells).

**Results:**

A genome scale network was constructed for *E. coli *K-12 by connecting genes with operons, transcription and sigma factors, metabolic pathways and cell functional categories. Most of the transcriptional changes were detected immediately upon entering lag phase and were maintained throughout this period. The lag period was longer for older cells and the analysis of the transcriptome revealed different intracellular activity in young and old cells. The number of genes differentially expressed was smaller in old cells (186) than in young cells (467). Relatively, few genes (62) were up- or down-regulated in both cultures. Transcription of genes related to osmotolerance, acid resistance, oxidative stress and adaptation to other stresses was down-regulated in both young and old cells. Regarding carbohydrate metabolism, genes related to the citrate cycle were up-regulated in young cells while old cells up-regulated the Entner Doudoroff and gluconate pathways and down-regulated the pentose phosphate pathway. In both old and young cells, anaerobic respiration and fermentation pathways were down-regulated, but only young cells up-regulated aerobic respiration while there was no evidence of aerobic respiration in old cells.

Numerous genes related to DNA maintenance and replication, translation, ribosomal biosynthesis and RNA processing as well as biosynthesis of the cell envelope and flagellum and several components of the chemotaxis signal transduction complex were up-regulated only in young cells. The genes for several transport proteins for iron compounds were up-regulated in both young and old cells. Numerous genes encoding transporters for carbohydrates and organic alcohols and acids were down-regulated in old cells only.

**Conclusion:**

Network analysis revealed very different transcriptional activities during the lag period in old and young cells. Rejuvenation seems to take place during exponential growth by replicative dilution of old cellular components.

## Background

In a laboratory environment, bacteria inoculated in batch culture grow until a maximum density is reached, after which they stop growing and start a cellular degenerative process that ultimately leads to death. The latter period is known as stationary phase and its duration depends on the environment. Cellular degeneration of bacteria in stationary phase has been found to reveal similarities with the aging process of higher organisms [1]. Senescence during stationary phase is followed by loss of ability to grow at the end of the stationary phase, which has been described as the nearest bacteria come to a "natural" death that occurs among aging organisms [1]. If stationary-phase bacteria are subcultured into fresh media, at a lower cell density, they will enter lag phase and halt the degenerative process, getting ready to start the division cycles again. The duration of the lag phase depends on the length of time the cells have spent in stationary phase before inoculation [2,3]; thus cells starved in stationary phase need a longer time to prepare for the first division.

Exponential growth rate can be predicted with a high degree of accuracy as a function of the current growth environment. Conversely, the duration of lag phase can be highly variable and single-cell studies have shown that it is influenced not only by the current growth environment but also by the previous history of the cells [4]. The molecular mechanisms underlying lag phase remain to be characterized. A major problem is that the low concentration of cells during lag phase makes it challenging to apply a number of analytical techniques. In yeast, a number of genes induced during lag phase are known to be involved in molecular biosynthesis and carbohydrate metabolism [5,6]. A rapid change in gene expression has also been detected in yeast populations entering lag phase [7]. These authors detected 2500 genes immediately up-regulated at the initiation of the lag phase and showed that this was because the RNA polymerase II was "poised" upstream of many inactive genes in stationary phase. In *Listeria monocytogenes*, experiments involving a few specific genes showed that the transcripts from a functional *sigB *gene were accumulated for an extended period during lag phase after an osmotic upshift [8] and, at low temperatures, lag phase was extended in the absence of a functional *sigB *gene [9]. In *E. coli*, chromatographic/mass spectrometry measurements have been optimized for the analysis of intracellular metabolites [10] and combined transcriptome and proteome analysis [11] has been carried out at high bacterial density during late exponential and stationary phase; however, such studies during lag phase are lacking and there is no comprehensive picture of the biochemical and molecular genetic activity of bacteria during this important period.

In our work, we have adopted a network-science approach to identify and interpret the differences at the transcriptional level between populations of young and old cells undergoing lag phase. The study of networks has a long tradition in graph theory and discrete mathematics, sociology, communication research, bibliometrics/scientometrics, webometrics/cybermetrics, in physics and, recently, in biology [12]. This approach has already been used to model the transcriptional regulatory network of *E. coli *[13], based on the publicly available network of *E. coli *MG1655, which contained originally 418 operons and 519 interactions. Protein-protein interactions in *E. coli *have also been identified and compiled in a large scale network [14,15]. More generally, the construction of metabolic networks in bacteria has been shown to be a very valuable tool to elucidate the components and pathways of biological processes [16-19].

In this paper, we have compared the lag-phase transcription profile of cells originated from an early stationary phase culture (young cells) with that of cells originated from a culture starved in stationary phase for 16 days (old cells). A genome scale network was constructed according to the metabolic pathways, functional roles, transcriptional regulation and predicted operon composition of *E. coli*. Our aim was to quantify and compare the complexity of the intracellular events during the lag phase of cells of different ages.

## Results and Discussion

### Quantitative microarray results

The time 0 h samples (cells in stationary phase) were used as the reference sample for gene expression analysis during the lag phases of old and young cells. Changes during lag phase in old (or young) cells are relative to the time 0 h sample of old (or young) cells. This type of transcriptomic experiment provides information on the relative levels of expression during the lag phase rather than a direct comparison of the absolute levels of gene transcripts between young and old cells. The number of genes that were significantly up/down-regulated with respect to stationary phase is reported in Table 1. In both old and young cells, the number of genes down-regulated during lag was greater than the number of genes up-regulated. In young cells, 296 genes were down-regulated in at least one sample, out of which 220 were down-regulated in all the samples. Conversely, 149 genes were up-regulated after 15 minutes in lag and 146 after 1 hour. From these, 127 genes were up-regulated after both 15 minutes and 1 hour. In old cells, 31 genes were up-regulated at all sampling times during lag, whilst 74 were up-regulated at least once. From 112 down-regulated genes, 67 were down-regulated in all samples. Data in Table 2 show that 41 genes were down-regulated in both old and young cells, whilst 21 genes were up-regulated in both.

**Table 1:** Number of genes up and down-regulated during lag phase with respect to stationary phase

	Young Inoculum						Old Inoculum					
	
Regulation	Sampling time during lag (min)				In all samples	Total	Sampling time during lag (min)				In all samples	Total
						
	15	30	45	60			75	150	225	300		
UP	149	153 (139)^1^	154(133)	146(127)	119	171	60	57(50)	56(45)	51(41)	31	74
DOWN	267	294(265)	267(242)	249(226)	220	296	105	92(86)	88(83)	91(87)	67	112

^1 ^The number of genes detected also as up(down)-regulated after 15 min in lag is between brackets

**Table 2:** Number of genes in each category according to their expression: down- or up-regulated or not modified, NM, during the lag phase of young and old cells

Cluster	Young cells inoculum	Old cells inoculum	Number of genes
1	Down	Down	41
2	Up	Up	21
3	Down	NM	296
4	Up	NM	170
5	NM	Down	112
6	NM	Up	74
7	Up	Down	1
8	Down	Up	2

Only three genes showed an opposite regulation response between old cells and young cells. The gene *sdhA*, a subunit of succinate dehydrogenase, was up-regulated in young cells and down-regulated in old cells. The genes *sufA*, whose product is involved in the biosynthesis of iron-sulfur clusters, and *yhaH*, a putative cytochrome, were down-regulated in young cells and up-regulated in old cells. Cluster analysis was carried out on the correlation matrix of the transcription profile of the genes up- and down-regulated during the lag phase (additional file 1: Fig S1). Expression patterns in time could not be identified. Some discontinuities in the expression patterns were assumed to be due to detection limits. There were no genes that were detected as both up- and down-regulated at different sampling times during lag, either in young cells or old cells.

### Analysis of the genome scale network and the sub-netwroks of genes up- and down-regulated during the lag time of young and old cells

**Figure 1:**
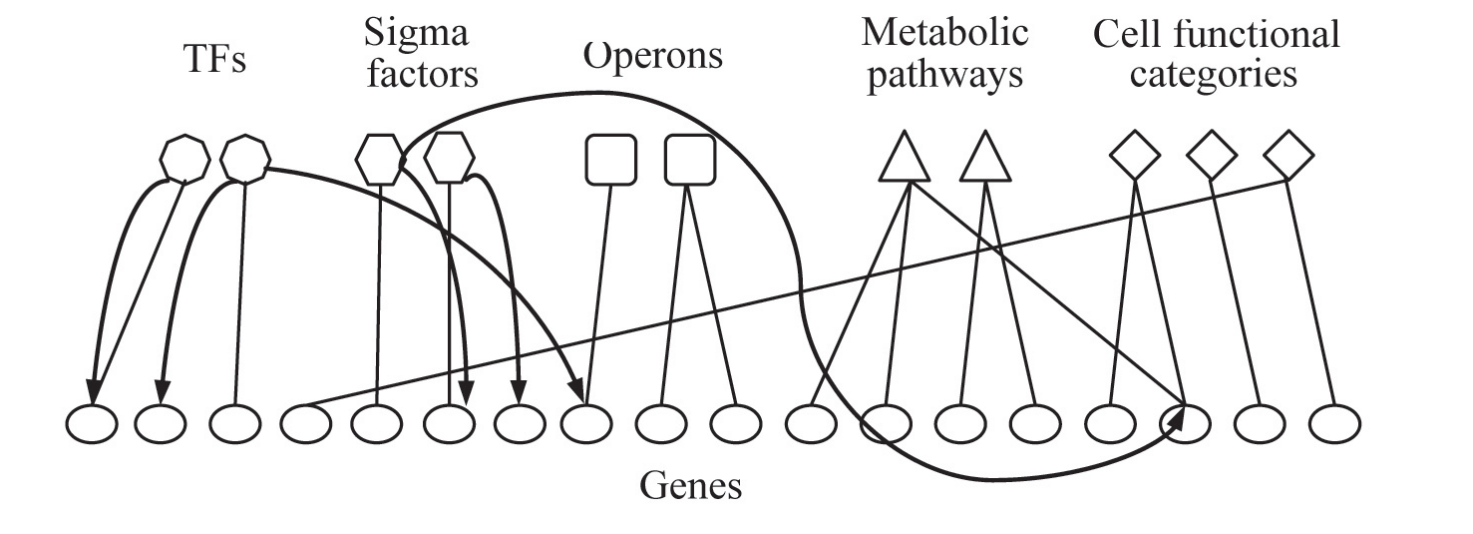
**Representation of a genome scale network for *E. coli *K-12**. The bipartite network contains genes (circles) connected to a set of nodes that include operons (squares), transcription factors (octagons), sigma factors (hexagons), metabolic pathways (diamonds) and cell functional categories (triangles). Edges connect gene nodes with the other node categories if associated. Arcs connect transcription and sigma factors with genes whose expression is regulated by them.

Fig 1 shows the bi-partite network constructed for the genome of *E. coli *K-12. Edges and arcs connect two sets of nodes. There are not direct connections between nodes of the same set. Genes constitute one set of nodes. The other set of nodes are divided into 5 categories: transcription factors (TFs), sigma factors, operons, metabolic pathways and cell functional roles. This structure was chosen to minimize the complexity and the size of the network. In addition, sub-networks pertaining to transcriptional regulation, operon structure, metabolism or cell functionality can be easily extracted by selecting the genes connected to the relevant category.

**Figure 2:**
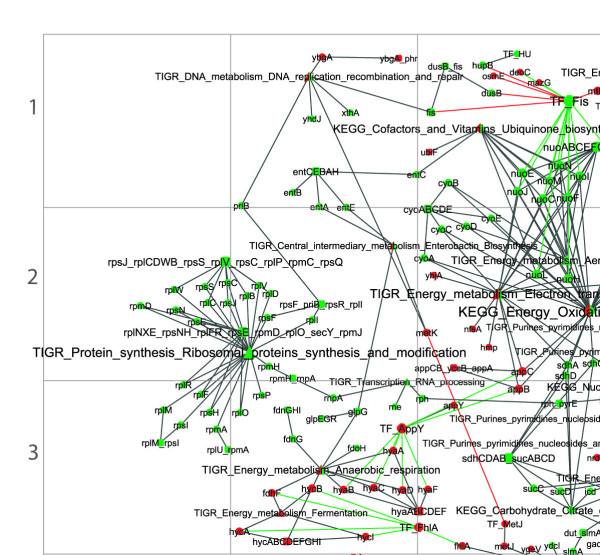
**Gene expression during lag phase of young cells**. Symbols as in Fig 1. Symbols are coloured in green if transcription was up-regulated and in red if down-regulated. Green (red) arcs connect transcription factors with genes whose transcription is initiated (repressed). Orange arcs connect sigma factors with the regulated genes. The sizes of nodes are proportional to their degrees. This figure shows the upper left quartile, for the full image please see additional file 4

**Figure 3:**
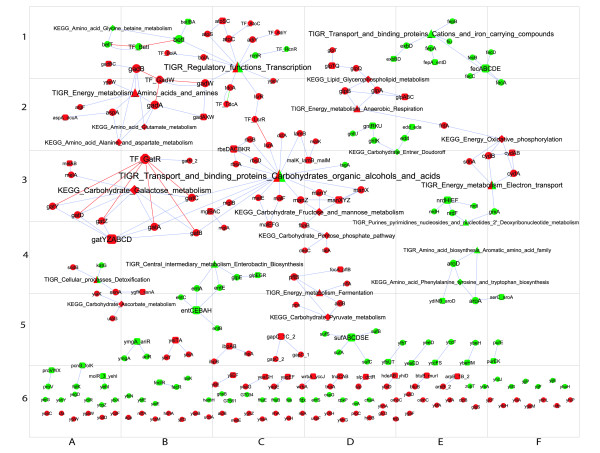
**Gene expression during lag phase of old cells**. Legend as in Fig 2.

The sub-networks corresponding to the genes up and/or down-regulated during the lag phase of old and young cells were extracted from the genome scale network and compared (Figs 2 and 3).

Table 3 shows the number of elements of the genome scale network and the sub-networks for old and young cells. In spite of all the information included in the genome scale network, ca. 20% of the genes were not connected to any operon, transcription factor, sigma factor, metabolic pathway or functional role. The percentage of unconnected genes was 32% in the network for young cells and 42% for old cells. Therefore, the number of genes with unknown function involved in the lag period is greater than expected, being remarkably large for old cells. There were some TFs differentially expressed in old and young cells; however, the number of arcs, i.e. genes regulated by those TFs, could be considered smaller than expected. The networks for old and young cells are based on detected gene expression. Therefore, they should not be expected to include all available information on transcriptional regulation since not all the genes known to be regulated by a TF will be detected under all conditions.

**Table 3:** Description of the genome scale network for *E. coli *and of the sub-networks of the genes differentially expressed during the lag time of old and young cells

Elements	Genome network	Young cells network	Old cells network
Total nodes	5768	656	266
Genes	4618 (80%)^1^	465 (71%)	185 (70%)
Operons	833 (14%)	130 (20%)	50 (19%)
Sigma factors	7 (0%)	3 (0%)	0 (0%)
TFs	159 (3%)	19 (3%)	9 (3%)
KEGG paths	57 (1%)	9 (1%)	11 (4%)
TIGR functions	94 (2%)	30 (5%)	11 (4%)
			
Unconnected genes	938 (20%)^2^	149 (32%)	73 (42%)
			
Total links	11032	612	77
Arcs	4812 (43%)^3^	74 (12%)	13 (16%)
Edges	6220 (57%)	538 (88%)	64 (84%)

^1 ^Percentage over the total number of nodes

^2 ^Percentage over the total number of genes

^3 ^Percentage over the total number of links

Connectivity, degree distribution, modularity and nestedness [20] were estimated on the genome scale network and compared with the sub-networks corresponding to genes differentially expressed in old and young cells. To assess the results, we analysed between 10 and 50 networks, with the same number of genes randomly selected.

The genome scale network had a giant connected component with 3645 nodes (63% of the total number of nodes) and 262 small disconnected components, each with a number of nodes between 2 and 11. The sub-network for young cells had a component connecting 43% of the nodes and numerous smaller components with less than 3% of nodes. The sub-network for old cells had two components connecting 20 and 8% of the vertices and smaller components with a percentage of nodes smaller than 5%. The random networks generated for young and old cells showed very similar connectivity patterns.

**Figure 4:**
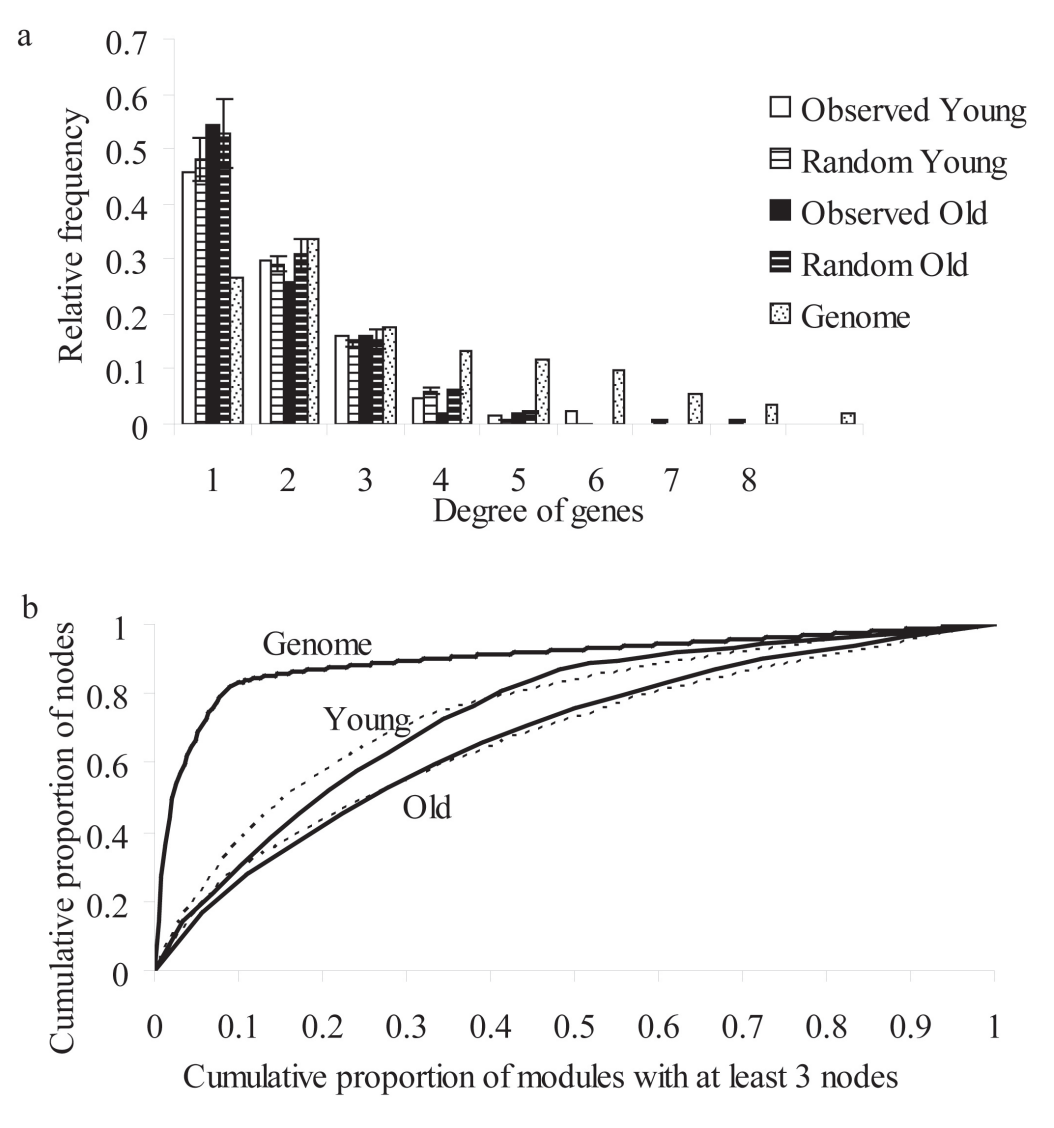
**Degree of genes and modularity of the networks**. (a) Distribution of degree of genes for the genome scale network and for the observed and randomly generated networks of genes differentially transcribed in old and young cells; (b) Distribution of the nodes in modules or communities for the genome scale network and the observed and randomly generated (dashed lines) networks of genes differentially transcribed in old and young cells.

In the genome scale network, the number of edges and arcs belonging to the same gene (degree of genes) did not seem to follow any of the commonly known degree distribution laws. The degree distributions of the other node categories were estimated independently for each category, and they followed the power law in every case, i.e. the sub-networks for these nodes belonged to the family of scale-free networks characterized by the presence of hubs or nodes connected to a large number of genes, as described previously [21]. For the old and young networks, the distribution of the degree of the genes was equal to that of the randomly generated networks (Fig 4a), while the number of nodes in the other categories was not enough to build distributions. Regarding the identity of the hubs or highly-connected genes in the genome scale network, the gene *gadA *had the highest degree (16), followed by *lpd, gabT, gltB, gltD, flhC, flhD, atoB, rpoH, cysG, nirB, nirD, aceE, aceK, galT, galE, paaF, paaG, fadJ, fadB, gadX, adhE *and *gadB*, all of them with a degree greater than 12. The products of these genes are mainly involved in lipid and amino acid metabolism, energy metabolism, chemotaxis and transcription. Two of these genes, *gadA *and *gadB *were also hubs in both young and old networks while the *rpoH*, and *fadJ *genes were differentially transcribed only in young cells. Assuming that the number of hubs extracted in the sub-networks has a hypergeometric distribution, the probability of finding 4 and 2 of the 23 hubs mentioned above in the network for young and old cells, respectively, is greater than 0.2. Regarding the other node categories in the genome scale network, 80 genes were connected to pyrimidine metabolism and 51 to purine metabolism. Between 40 and 47 genes were connected to the pathways of glycolysis, oxidative phosphorylation and fructose and mannose metabolism. The cell functional categories with the highest number of links were regulatory functions (173), transport and binding proteins for carbohydrates, organic acids and alcohols (103) and (respectively) for amino acids, peptides and amines (94); and DNA replication recombination and repair (90). Most of these metabolic pathways and functional roles were differentially expressed in both young and old cells during lag.

Modularity was estimated with the program implementing the fast modularity maximization algorithm [22]. Networks have communities of highly interconnected nodes that are less connected in other modules and this organization in communities is quantified by the modularity value, Q. If the fraction of within-community edges is no different from what we would expect for a randomized network, then Q will be zero. Nonzero values represent deviations from randomness, and in practice it is found that a value above about 0.3 is a good indicator of significant community structure in a network [22]. The value of Q was 0.6 for the genome scale network while the sub-networks of young and old cells showed a greater modularity, with values of 0.87 and 0.91, respectively. These modularity values were not different from those generated for the correspondent randomly generated networks. Fig 4b shows the distribution of nodes in modules. The genome scale network had 231 modules with more than 3 nodes each. From these, 10% of the modules included 80% of the nodes (Fig 4b). The networks for young and old cells showed a larger relative number of modules, 40% and 60%, respectively, containing 80% of the nodes. These distributions were not different from the correspondent randomly generated networks (Fig 4b) and therefore the greater level of modularity is due to the smaller numbers of nodes.

Nestedness is a key feature to explain the level of organization of bipartite networks [20]. Nestedness comprises a non-random pattern in which the most connected set of elements of both classes interact amongst themselves, generating a dense core of interactions to which the rest of the elements are attached. To calculate the level of organization or nestedness, we used the nestedness calculator program [23]. Here, nestedness is defined in the interval [0, 1], where 1 corresponds to a perfectly nested network. The levels of nestedness between genes and TFs, sigma factors, KEGG pathways and TIGR cellular roles were calculated independently for each category. To compare the result between the different categories, we calculated the ratio between the nestedness value of the matrix and the nestedness coefficient obtained by Monte-Carlo simulation from randomly generated matrices. If this ratio was significantly greater than 1, the network was considered to show a nested pattern. Table 4 shows that only the sigma factors and TFs showed a significant nested pattern in the genome scale network. This cohesive pattern can provide alternative routes by which the transcriptional network can respond to perturbations. In the networks of gene expression for old and young cells, nestedness was not detected for any node category. Therefore, the detected TFs did not show a nested structure with the differentially expressed genes. As mentioned above, it should not be expected that all genes regulated by a TF or sigma factor will be detected in response to all stimuli.

**Table 4:** Nestedness (N) quantification for the genome scale network and for the sub-networks of differentially expressed genes during the lag phase of old and young cells

	Genomic network				Young cells network				Old cells network		
	Sigma factors	TFs	KEGG paths	TIGR functions	Sigma factors	TFs	KEGG paths	TIGR functions	TFs	KEGG paths	TIGR functions
N	0.897	0.985	0.911	0.884	0.495	0.829	0.609	0.803	0.490	0.574	0.665
Nr ^1^	0.588	0.942	0.908	0.967	0.779	0.862	0.657	0.911	0.776	0.827	0.830
Ratio (N/Nr)^2^	1.526	1.046	1.003	0.914	0.635	0.963	0.927	0.881	0.632	0.694	0.801
*p *value^3^	< 0.0001	< 0.0001	0.122	1.000	1.000	0.911	0.889	1.000	0.992	1.000	1.000

^1^Nestedness obtained by Montecarlo simulation from 50 matrices randomly generated

^2^Ratios are greater than 1 if nestedness is greater than randomly expected

^3^Probability that the nestedness coefficient is equal to or less than that randomly generated

Therefore, the most relevant topological features of the genome scale network either remain constant in the sub-networks of the genes differentially expressed in old and young cells or, should they change, this change is also detected in the correspondent randomly generated network.

### Comparing transcriptional activity during the lag phase of young and old cells

Changes in gene expression were difficult to differentiate from failure in detection for samples obtained at different time points during the lag period as seen by cluster analysis (additional file 1: Fig S1). Therefore, the transcriptional regulatory network and predicted operons, detected as up- and/or down-regulated in both young and old cells, were in general not affected by the sampling time during lag (additional file 2: Fig S2 and additional file 3: Fig S3). Neither the regulation of metabolic pathways and functional roles varied through the lag period. Most of the transcriptional changes were detected on entering lag phase and were maintained throughout this period. For this reason, the sampling time during the lag period was not taken into account for the analysis.

**Figure 5:**
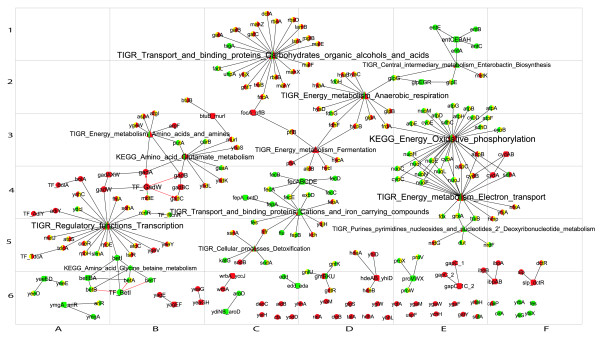
**Genes affected during lag phase of both old and young cells**. Symbols as in Fig 1. The right half of the node represents the result in old cells and the left half in young cells: green if up-regulated and red if down-regulated.

**Figure 6:**
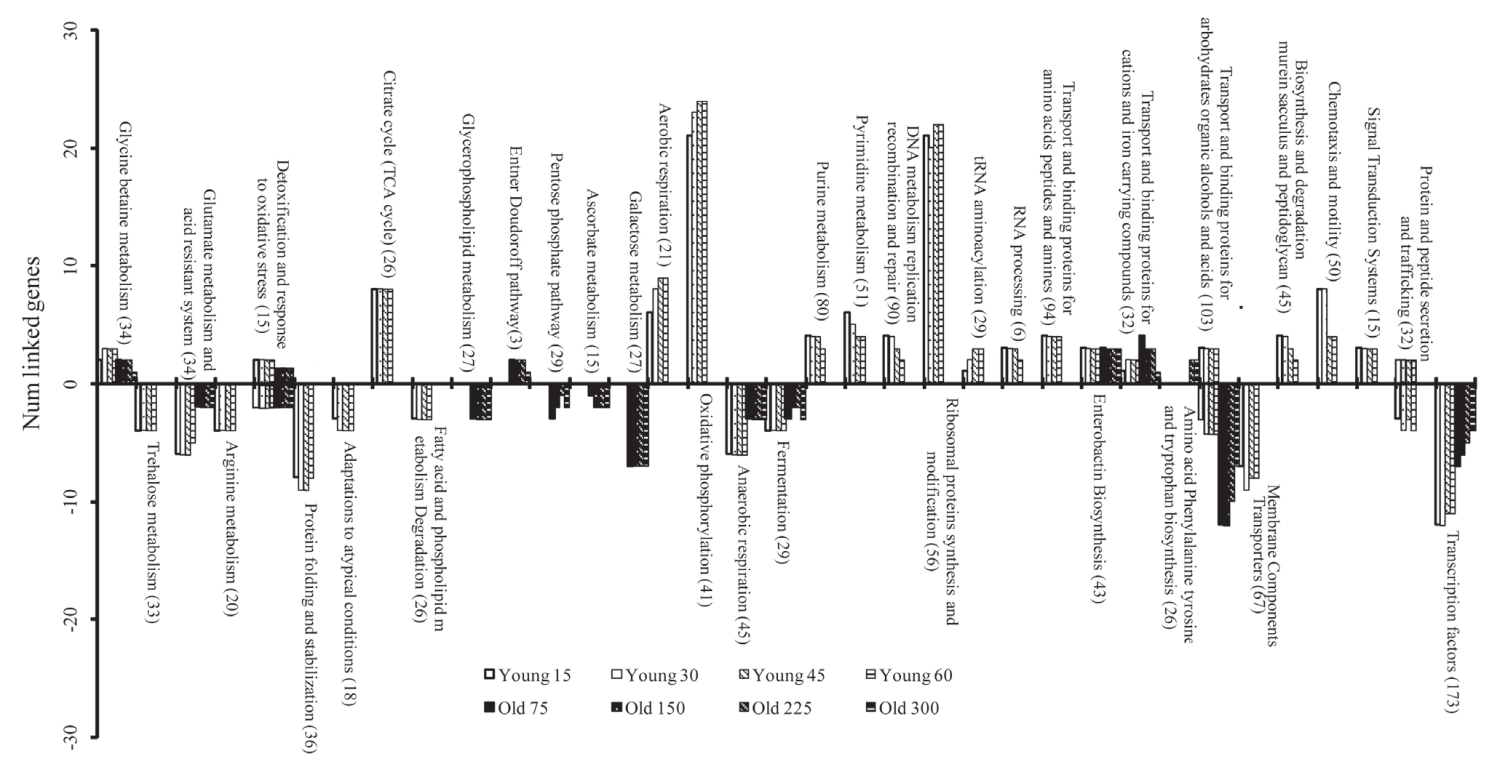
**Metabolic pathways and cellular roles with a significant (*p *< 0.1) proportion of genes up- or down-regulated in young or old cells during the lag phase**. Sampling times in minutes.

Figs 2 and 3 show the sub-networks of the genes up- and down-regulated during the lag phase in young and old cells, respectively. Fig 5 shows the intersection of the networks in Figs 2 and 3, i.e. genes differentially expressed in both old and young cells. Fig 6 shows the metabolic and functional roles detected as significantly up- or down-regulated during the lag of young and old cells. Fig 6 will be used as an index to explain the results and explore the network maps. In the following sections, the transcriptional responses are categorised and analysed according to physiological function and/or biochemical mechanism, commencing with stress responses.

#### Osmotolerance

The mechanisms of osmotolerance are not fully understood. Bacteria utilize intracellular compatible osmolytes such as glutamate, betaine, proline, and trehalose to adapt to osmotic stress arising from extracellular solutes [24,25]. Upon entering lag phase, both young and old cells up-regulated several elements of the glycine-betaine osmoprotective system (Fig 5, 5B). The *betA*, *betB *and *betT *genes and the BetI TF, which regulates the transcription of the system, were all up-regulated in young cells (Fig 2 and additional file 4, 4D) and two of them, the *betT *gene and BetI TF, were up-regulated also in old cells (Fig 3, 1A). In addition, genes from the *proVWX *operon for the transport of glycine-betaine were up-regulated in both cultures (Fig 5, 6E). Regarding the metabolism of proline, the genes encoding the PutP transport protein for proline uptake and the PutA TF that represses proline catabolism genes [26] were up-regulated in young cells only (Fig 2 and additional file 4, E3, 1F). These changes are perhaps surprising given that the inoculation of stationary phase cells into fresh medium did not impose an osmotic stress. It is likely that the change in the medium afforded cells the opportunity to modify the mechanism to contend with the osmotic potential of the medium. *E. coli *is able to synthesize glutamate, betaine, proline, and trehalose during oxidative metabolism provided that choline is available, but biosynthesis is limited to glutamate and trehalose in anaerobic conditions such as stationary phase [24,25]. In young cells, but not in old cells, glutamate metabolism and transport (Fig 2 and additional file 4, 2E), as well as the signal transduction complex to sense extracellular osmotic stress and synthesize large amounts of trehalose (Fig 2 and additional file 4, 4E), were active during stationary phase and were down-regulated when cells were inoculated in fresh medium at a lower cell concentration. The same pattern was observed with TreR, the TF that regulates the latter system. High cell density in a batch culture in stationary phase causes an anaerobiosis situation that reverts when cells are inoculated at lower cell concentration into fresh medium.

#### Acid resistance

Three known systems have evolved for stationary-phase acid resistance in *E. coli *[27]. The acid resistance system 1 requires the sigmaS subunit of the RNA polymerase, RpoS, which is the master regulator of the general stress response [28]. This sigma factor was not differentially transcribed upon entrance into lag phase either in old or young cells. The acid resistance system 2 was down-regulated in both young and old cells. This system is glutamate-dependent and it couples the transport activity of the GadC protein with a glutamate decarboxylase, GadA/GadB. The genes encoding these proteins are part of the operons *gadAXW *and *gadBC *and were down-regulated during the lag phase of both young and old cells. The GadW TF that represses the expression of this system was also down-regulated in both cultures. The last system is arginine-dependent and is based on the arginine decarboxylase and the AdiC antiporter to exchange extracellular arginine with agmatine, which is the product of arginine decarboxylation. The genes encoding the AdiC antiporter and the ArtJ and ArtP proteins for arginine uptake, together with the *argF*, *argG*, and *argI *genes associated with arginine biosynthesis from glutamate, were all down-regulated in young cells only. However, the gene for the adiY TF that controls the arginine decarboxylase system under anaerobic conditions [29] was down-regulated in both young and old cells. The observed pH values were of *ca. *6 and ca. 7 after 1 day (young cells) and 16 days (old cells) in stationary phase, respectively. Although these pH values do not imply acid stress, several responses to acid stress were activated during the stationary phase. It has been already shown that defence systems against acid stress respond positively to stationary phase, and its induction does not require external acid pH [30,31]

#### Oxidative stress

Three distinct superoxide dismutases (SOD) have been described in *E. coli*: the manganese-SOD, encoded by the *sodA *gene, detected in aerobiosis and mainly responsible for preventing damage to DNA, the iron-SOD encoded by the *sodB *gene, detected in both anaerobiosis and aerobiosis and mainly responsible for protecting cytoplasmic enzymes [32] and a copper-zinc-SOD found in a mutant unable to produce any of the previous two enzymes [33]. Upon entering lag, both old and young cells down-regulated the gene encoding iron-SOD (Fig 5, 5C). The gene for manganese-SOD was up-regulated only in young cells, while the gene for the Rob TF that activates its transcription was down-regulated (Fig 2 and additional file 4, 4D). These changes could be a consequence of anaerobiosis associated with high cell density in stationary phase. On the other hand, the *katG *gene encoding hydroperoxidase I, which functions as both a catalase and a broad spectrum peroxidase [34], was up-regulated in both young and old cells in lag phase (Fig 5, 5C). Therefore, the strategy to contend with the redox potential of the medium changed when cells entered lag period.

Oxidative damage has been associated with cellular senescence in *E. coli *in stationary phase [1]. Heat shock proteins have been linked to a possible role in counteracting protein oxidation in *E. coli *cells in stationary-phase cells [1]. These proteins have been shown to mitigate starvation-induced protein carbonylation, which is an irreversible modification associated with senescence [35]. The heat shock response in *E. coli *is controlled by two distinct RNA polymerase species in *E. coli*: the sigma E or 24 and sigma H or 32 factors [36,37]. In young cells, the genes encoding both these factors, as well as several genes whose transcription is regulated by them, were down-regulated (Fig 2 and additional file 4, 4B, 5B). The sigma E factor is regulated by a multistep protease system that senses protein disruption [38]. Its transcriptional initiation targets include the UspA and UspD proteins and the Sigma 32 factor, all of them detected as down-regulated in young cells (Fig 2 and additional file 4, 4B, 5B). The UspA and UspD proteins are universal stress response regulators recently linked to oxidative stress defence and important in delaying cell senescence [1,39]. The Sigma 32 factor regulates the DnaK/DnaJ/GrpE, IbpA/IbpB and GroEL/GroES chaperone systems, which were all down-regulated in young cells (Fig 2 and additional file 4, 4B, 5B). These systems assist protein folding and stabilization and prevent aggregation of misfolded proteins [40]. The DnaK/DnaJ and GroEL/GroES systems are strongly involved in defence against oxidative stress [1]. Therefore, this defence mechanism against oxidative damage to proteins was down-regulated in young cells but not in old cells. It would appear that old cells keep this system active even after encountering an optimum environment or may deactivate this mechanism after some time in stationary phase. The *wrbA *gene, encoding a quinone oxidoreductase involved in resistance to oxidative stress [41], was down-regulated in both cultures (Fig 5, 6C).

#### Other environmental stresses

Several genes induced by the Sigma E factor and involved in adaptation to and/or defence against stress conditions were also down-regulated in young cells (Fig 2 and additional file 4, 4B). These included the gene for the CspD protein that inhibits chromosome replication in stationary phase and the *lpxP *gene demonstrated to respond to cold shock conditions. A number of genes for membrane transporter systems with detoxification activities were down-regulated in young cells only. These were the EvgA TF (Fig 2 and additional file 4, 3E), associated with acid and multidrug resistance, and components of the MdtEF-TolC multidrug efflux transport system (Fig 2 and additional file 4, 3E) and of some transporters from the Rhs family (Fig 2 and additional file 4, 2F) reported to prevent toxicity in stationary phase [42]. Some genes related to stress response were also down-regulated in old cells after inoculation in fresh medium. The *slp-dctR *operon that encodes starvation lipoproteins was down-regulated in both young and old cells (Fig 3, 6F).

Therefore, responses to several stresses were down-regulated upon transfer to improved growth conditions. The number of genes related to stress response in stationary phase and down-regulated in young cells was larger than in old cells. This could indicate that the stress response is activated at the beginning of the stationary phase and partially deactivated later.

#### Carbohydrate catabolism and energy generation

*E. coli *is capable of three alternative modes of energy generation: aerobic respiration, anaerobic respiration and fermentation [43]. During lag phase, young cells appeared to begin aerobic respiration, whereas in old cells there was no evidence of aerobic respiration. In both old and young cells, anaerobic respiration and fermentation pathways were down-regulated after inoculation into fresh medium.

Practically all genes of the main operons encoding the enzymatic complexes involved in aerobic oxidative phosphorylation were up-regulated in young cells but not in old cells. These operons were those encoding NADH dehydrogenase and its transcription regulator, Fis protein (Fig 2 and additional file 4, 1C), fumarate reductase/succinate dehydrogenase (Fig 2 and additional file 4, 3CD), cytochrome oxidase (Fig 2 and additional file 4, 2C) and ATP synthase (Fig 2 and additional file 4, 1D). However, in old cells, the *sdhA *gene that encodes a component of one of these enzymes, succinate dehydrogenase (SdhCDAB), associated with aerobic respiration, was down-regulated (Fig 3, 3E). In young cells, although genes encoding the SoxR and Rob TFs that activate the transcription of the aerobic fumarase C were down-regulated, the gene, *fumC*, encoding that enzyme was nevertheless up-regulated (Fig 2 and additional file 4, 4D). Other TCA cycle genes up-regulated in young cells were those of the *sdhCDAB-sucABCD *operon, encoding the already-mentioned succinate dehydrogenase and the succinyl-CoA synthetase, and the *icd *encoding for the isocitrate dehydrogenase (Fig 2 and additional file 4, 3C). Also in young cells only, the gene encoding the UhpA TF, promoting the uptake of exogenous hexose phosphates [44], was up-regulated (Fig 2 and additional file 4, 3F).

Two genes, *cydA *and *cydB*, expressing the subunits of the cytochrome bd-I terminal oxidase, were down-regulated in old cells (Fig 3, 3F) and one of them, the *cydA *gene, was also down-regulated in young cells (Fig 2 and additional file 4, 2D). This enzymatic complex has been associated with aerobic respiration under microaerobiosis [45], which would explained its role during stationary phase, followed by down-regulation when cells encounter aerobic conditions after inoculation in fresh medium at lower cellular concentration. Regarding anaerobic metabolism, the expression of the *hya *operon and its transcription regulator, AppY, were down-regulated in young cells (Fig 2 and additional file 4, 3B). This operon encodes the hydrogenase 1 and it is induced under anaerobic conditions and by the presence of formate [46]. The *frdA *gene from the fumarate reductase complex (FrdABCD), which acts most effectively under anaerobic conditions [47] and the genes encoding the anaerobic dehydrogenase protein complex, GlpABC, were down-regulated in old cells (Fig 3, 2DE). Upon entering lag phase, young cells repressed the transcription of the enzymatic complex FadI/FadJ that catalyzes the beta oxidation of fatty acids and allows *E. coli *to use this compounds as carbon and energy source under anaerobic conditions [48], as well as of the CaiF TF that activates the transcription of enzymes involved in the metabolism of carnitine, which is essential for fatty acid metabolism and transport [49] (Fig 2 and additional file 4, 1F). In old cells, the genes *glpB*, *glpA*, *glpQ *and *glpT*, encoding enzymes for the anaerobic metabolism of glycerol and glycerophospholipids, were down-regulated (Fig 3, 2D).

During fermentative growth, glycolytic carbon sources are converted to pyruvate, and thence to acetyl-CoA and formate by the activity of the pyruvate formate-lyase, PflA, enzyme [43]. Upon entering lag phase, young and old cells down-regulated this enzyme (Fig 5, 3C). The resulting formate may then be either excreted, by the FocA formate transporter, or further metabolized by the formate hydrogenlyase, Fhl-1, system. The genes required for the synthesis of Fhl-1 system form the formate regulon, which includes three transcriptional units, namely, the *hycABCDEFGHI *and *hyp-fhlA *operons and the *fdhF *gene [43]. The gene encoding the FocA transporter (Fig 2 and additional file 4, 3E) and the three transcriptional units of the formate regulon (Fig 2 and additional file 4, 3B) were all down-regulated in young cells.

Changes in carbohydrate metabolism in old cells were related to the Entner Doudoroff and gluconate pathways. The Entner Doudoroff pathway involves gluconate and its degradation [50]. The *edd *gene from the Entner Doudoroff pathway, together with genes for the GntU gluconate transporter and for the gluconate kinase enzyme, encoded by the *gntK *gene and involved in gluconate degradation, were up-regulated in old cells (Fig 3, 3E). In young cells, the *edd *gene from the Entner Doudoroff pathway was also up-regulated, but genes related to gluconate degradation were not detected and furthermore the genes for the gluconate transporter, GntT, and for the transcription regulator of this system, the GntR TF, were down-regulated (Fig 2 and additional file 4, 3F). Intracellular gluconate can also be obtained via the pentose phosphate pathway. In old cells, genes encoding enzymes of the pentose phosphate pathway were down-regulated (Fig 3, 4D). In addition, the L-ascorbate degradation pathway was also down-regulated (Fig 3, 5A); this pathway results in products that can enter the non-oxidative branch of the pentose phosphate pathway [51]. Therefore, the pentose phosphate pathway seems to be active in old cells in stationary phase but it is deactivated upon entering lag phase. Similarly, galactitol metabolism was down-regulated in old cells, because the gene for galactitol permease, together with several genes related to its degradation and also the GatR TF which regulates transcription of the system, were all down-regulated (Fig 3, 3B).

#### Macromolecule biosynthesis

Fig 6 shows that a number of genes associated with salvage pathways, as well as with pathways for the *de novo *biosynthesis of pyrimidine and purine nucleotides, were up-regulated during the lag phase of young cells only (Fig 2 and additional file 4, 2D, 3D), together with genes encoding several proteins related to DNA metabolism. These were the bacterial histone-like HU protein involved in DNA compaction, the XthA protein, related to the organization and maintenance of nucleotide structure, the PriB protein, involved in replication, and the Fis protein (Fig 2 and additional file 4, 1B, 1C). The primary role of Fis is to maintain the structure of the genome[52]. Fis also regulates the transcription of a large number of genes involved in a variety of functional roles [53]. Several genes regulated by Fis and related mainly to aerobic respiration were also up-regulated only in young cells, as already mentioned. The regulation of genes encoding proteins associated with genomic DNA methylation essential for cell division [54] was variable; whilst the gene encoding the YhdJ protein was up-regulated (Fig 2 and additional file 4, 1B), those encoding the MetJ TF and MetK protein were down-regulated (Fig 2 and additional file 4, 2C, 3C) in young cells.

Translation and protein biosynthetic activities were only up-regulated in young cells. Thus, genes upregulated included those encoding several proteins involved in the biosynthesis of both the large and the small ribosomal subunits (Fig 2 and additional file 4, 2B); the *valS*, *glyQ *and *tgt *genes, encoding products for the aminoacylation (Fig 2 and additional file 4, 5D) of tRNA, and genes encoding ribonucleases for processing of tRNA, rRNA and mRNA (Fig 2 and additional file 4, 3B).

#### Transport functions

Transcription of genes encoding transport proteins for amino acids, peptides and amines was variable in young cells, while in old cells this function was not significantly affected. The PotABCD transport system for polyamines, putrescine and spermidine was up-regulated in young cells (Fig 2 and additional file 4, 1E). Lack of polyamines is associated with abnormal growth and oxidative stress-induced damage [55]. As mentioned above, genes for the transporters for glycine-betaine, ProXVW, and proline, PutP, were up-regulated, while the transcription of the gene for the AdiC antiporter for arginine, together with transcription of the genes for other transport proteins associated with acid and osmotic stress, was down-regulated in young cells (Fig 2 and additional file 4, 1E).

The genes encoding several transport proteins for cations and iron compounds were up-regulated in both young and old cells, such as the *entCEBA *operon involved in the biosynthesis of the enterobactin siderophore from chorismate (Fig 5, 1E). The *aroA *and *aroD *genes involved in chorismate biosynthesis were up-regulated in old cells only (Fig 3, 4E). Chorismate is also an intermediate metabolite required for the biosynthesis of the aromatic amino acids, phenylalanine, tyrosine and tryptophan. The gene encoding the outer membrane receptor for iron transport, Fiu, and the *fecABCDE *and *fepA-entD *operons related to iron transport and metabolism were up-regulated in both young and old cells (Fig 5, 4D). However, on entering lag phase, young cells down-regulated the *bfr *gene encoding the iron-storage protein, bacterioferritin, and the gene encoding the FeaA iron transport protein (Fig 2 and additional file 4, 5C).

The genes encoding numerous transporters for carbohydrates and organic acids and alcohols were down-regulated in old cells, whereas in young cells a smaller number of transport systems were affected. Systems down-regulated in old cells included those encoding for galactitol, GatABC, and mannose, ManXYZ, permeases, as already mentioned, and the *rbsDACBKR *operon encoding the ribose transporter. Also down-regulated in old cells were the genes encoding the DctA protein required for dicarboxylate transport, the LamB for diffusion of maltodextrins, components of the MalKFGE maltose transport system, the MglB component of the galactose transporter and the LsrA uncharacterized sugar transporter [42] (Fig 3, 3C). In young cells, the transport systems down-regulated included the genes for the already-mentioned gluconate and formate transporters, GntT and FocA, and trehalose permease, TreB. The genes encoding the long-chain fatty acid transporter, FadL, and the UhpA TF for the uptake of hexoses phosphates, as explained above, were up-regulated (Fig 2 and additional file 4, 3E). Both in old and young cells, the gene encoding the TsgA protein, a member of the major facilitator superfamily of transporters of metabolites, was up-regulated (Fig 5, 1C).

#### Cell-envelope components

Genes related to the biosynthesis of cell envelope components were up-regulated in young cells only. These included the *mraY*, *mrdB *and *dacB *genes involved in peptidoglycan biosynthesis and the *accD *gene, encoding a sub-unit of acetyl-CoA carboxylase, which catalyzes an initial step in the biosynthesis of phospholipid components of the cell membrane (Fig 2 and additional file 4, 3E). However, the *murI *gene that encodes the glutamate racemase involved in peptidoglycan biosynthesis was down-regulated (Fig 2 and additional file 4, 3E). Similarly, the *tar-tap-cheRBYZ*, *motAB-cheAW *operons *and flgK *and *fliD *were up-regulated only in young cells (Fig 2 and additional file 4, 5E). These genes encode structural components of the flagellum and several components of the chemotaxis signal transduction complex involved in the transmission of sensory signals to the flagellar motors that affect swimming behaviour (Fig 2 and additional file 4, 4E) [42].

#### Secretory pathways

The 'general secretory pathway', GSP, and its associated secreton complex, Sec, is used by most proteins that are completely translocated across the inner membrane and end up in the periplasm or outer membrane [56]. The genes encoding the SecD and SecF elements of the secretion complex were up-regulated in young cells. Conversely, the *gspM *and *gspC *genes that are similar to those coding for the main terminal branch of the general secretory pathway in *Klebsiella oxytoca *[57], together with those encoding other putative transporters, were down-regulated in young cells (Fig 2 and additional file 4, 5E).

#### Transcription factors

Upon entering lag phase, the gene encoding the BolA TF, associated with the maintenance of cell morphology in stationary phase, was down-regulated in both old and young cells. Numerous other TFs were down-regulated in both old and young cells. Thus, the genes for seven out of a total of nine TFs that were differentially expressed in old cells were down-regulated and, similarly, 16 out of a total of 21 were down-regulated in young cells (Fig 5, 5B). Some genes regulated by these TFs were differentially expressed but their expression did not always agree with the expected regulation. As an example, from the 173 genes regulated by Fis, 10 and 2 genes were, as expected, up- and down-regulated, respectively; whereas the *dusB *and *hupB *genes that were expected to be down-regulated by Fis were up-regulated and the *mazG *and *deoC *genes theoretically up-regulated by Fis were down-regulated (additional file 2: Fig S2). This is to be expected since static views provide poor quality maps of the transcriptional network, which is the result of precise expression timing and subsequent refinements. Apart from the already-mentioned TFs, the genes encoding the CueR TF, related to copper homeostasis, and the MtlR TF, for mannitol utilization, were only down-regulated in young cells. Conversely, the genes encoding the LsrR TF, involved in quorum sensing, the TdcA TF, for transport and metabolism of threonine and serine during anaerobic growth [58] and the AtoC TF, related to short-chain fatty acid metabolism, were all down-regulated only in old cells. The gene encoding the RcnR protein that regulates nickel, cobalt and iron homeostasis [59] was up-regulated during the lag of old cells only.

## Conclusion

The genome scale network built for *E. coli *provided an insight into the molecular responses occurring during lag phase, in relation to the duration of the immediately-preceding stationary phase. Information related to component systems, signalling, metabolic pathways, transcriptional control and specific cellular activities was integrated in a genome-scale partially directed network. This network met the important goal of handling different layers of information at the genome scale that otherwise would have required a great human and computing effort.

Stationary phase is a reversible process, such that if the growth-limiting factor is removed (i.e. the overpopulation condition and the chemical environmental changes associated with it), stationary phase cells are able to resume growth. Thus, stationary phase cells responded quickly to the new growth conditions by adjusting their transcriptional activity immediately after inoculation in fresh medium. Fig 2 and additional file 4 show how the network of genes differentially transcribed in young cells had a larger number of nodes and connections than the network for old cells. A greater transcriptional activity might be anticipated in old cells as a mechanism to counteract senescence during stationary phase. In addition, similar intracellular activities might be envisaged by the end of the lag period in both old and young cells. However, lag time does not seem to be the period in which senescence is wholly reversed; rather, during lag phase, cells prepare to start the division cycle by adopting different metabolic strategies according to their initial condition. In old cells, this process is longer and less efficient than in young cells, as shown by the lack of up-regulation of aerobic respiration, possibly because of the damage associated with aging. Rejuvenation may, effectively, take place later, during the exponential growth phase. Replicative rejuvenation during exponential phase is the converse of the process described as replicative senescence. Bacterial cells have been shown to exhibit signs of replicative aging, or loss of fitness, in a sibling-specific manner during exponential growth; i.e., a cumulative loss of fitness in sibling lineages that inherit old cellular poles [60]. However, and conversely, the same mechanism may in fact be contemplated equally as a replicative cellular rejuvenation process. A population starting from one single cell after *m *successive generations will include 2^*m*-1 ^cells (half the population) with structures formed during the last, *m*, and last but one, *m*-1, division cycles. Less than 1/4 cells of the population will have structures formed in division cycles previous to the *m*-2th division. In fact, only two cells will have the poles of the original single cell. Therefore, the findings described previously [60] can be read as conferring a growth advantage on cells with newly-formed poles; and furthermore, such cells represent the vast majority of the population. During exponential growth phase, the successive division cycles will have a dilution effect on the old and/or damaged cellular structures previously accumulated during senescence and will hence ensure the rejuvenation of the population.

## Methods

### Bacterial strain and growth conditions

*E. coli *K-12 substrain MG1655 was grown at 25°C for 48 hours (young culture) and 17 days (old culture) in Luria-Bertani broth (10 g/l Tryptone, 5 g/l yeast extract and 10 g/l NaCl; pH 7.2) with 0.2% glucose. Cultures were grown statically and reached stationary phase after ca. 24 h; therefore the time spent in stationary phase was ca. 1 and 16 days, for young and old cells, respectively. The time in stationary phase is considered as the age of the population, as it approximates the time elapsed from the last division for most of the cells in the population. These cultures were used to inoculate aliquots of 750 ml of fresh medium to give an initial concentration of 10^5^-10^6 ^cells/ml. The lag phases were ca. 1.5-1.8 and 4.7-5.1 hours for cultures inoculated with young and old cells, respectively. Samples were obtained for gene expression analysis at 0, 0.25, 0.5, 0.75 and 1 hours, and at 0, 1.25, 2.5, 3.75 and 5 hours after inoculation of the young and old culture, respectively. At each sampling time, the 750 ml culture was harvested by adding 1/5 volume of 5% phenol 95% ethanol (v/v) and placing the flask on ice for 30 minutes [61]. The cellular material was then pelleted by centrifugation at 6000 × *g *at 4°C for 15 minutes, with the pellet stored at -80°C until RNA isolation was carried out. Cell concentration was monitored by viable counts on Tryptone soy agar. The whole experiment was repeated twice.

### Construction of DNA microarrays

The *E. coli *K-12 MG1655 DNA microarrays used in this work were produced as described previously [61], with additional features added. Each microarray included approximately 100 features of serially diluted chromosomal DNA (15-20 replicates of each dilution) isolated from the same strain used for the microarray design (MG1655). These features are referred as genomic controls and used in data analysis.

### RNA and DNA purification and microarray hybridizations

RNA was purified from *E. coli *as described previously [62]. RNA quality and quantity was checked using the Agilent 2100 Bioanalyzer (Agilent Technologies). Genomic DNA was isolated using the QIAgen DNeasy™ method (QIAgen) following the manufacturer's instructions. Genomic DNA was labelled with Cy3-dCTP (Amersham) using a protocol based on the BioPrime labelling kit (Invitrogen), whilst RNA was labelled with Cy5-dCTP (Amersham) using Stratascript Reverse Transcriptase (Stratagene). The labelled cDNA and DNA were mixed together and competitively hybridized on a microarray slide overnight at 62°C. Following hybridization, microarray slides were washed and scanned using an Axon GenePix 4000A Microarray scanner (Axon Instruments, CA) and the feature intensities were quantified using GenePix Pro software (Molecular Devices). Full labelling, hybridisation and washing protocols are available on the IFR Microarrays web-site.

### Microarray data analysis

Data normalization and analysis was carried out using the program ArrayLeaRNA, which implements a Bayesian inference method based on genomic controls and operon transcription pattern [63]. Fluorescence intensities detected in the samples during lag were compared with the intensities of the sample obtained in stationary phase used as the inoculum (time 0). Only genes detected as up- (down-) regulated at least once in each replicated experiment carried out either with young or old cells were considered as differentially expressed. Three genes that were detected as both up- and down-regulated during the lag of young cells were deleted from the analysis.

### Genome scale network construction

A bi-partite network was constructed for the genome of *E. coli *K-12 as follows. Edges/arcs connected two sets of nodes. Genes constituted one of these sets of nodes. The genome composition was obtained from the EcoCyc database [42]. The other set of nodes included 5 categories: transcription factors; sigma factors and operons, as denoted in the RegulonDB v 6.0 database [64]; metabolic pathways, according to the KEGG database [65]; and functional role categories, as described in the CMR-TIGR database [66]. The information was completed and revised according to the Ecocyc database [42].

For network representation and topological quantification we used the programs PAJEK [67] and Cytoscape [68]. Networks modularity was estimated with the program implementing the fast modularity maximization algorithm [22]. The level of nestedness organization of the networks was estimated with the nestedness calculator program [23].

### Statistical test on the significance of the changes on expression of metabolic pathways, cell functional categories and operons

For an observed sample, the statistical evaluation of the up(down)-regulation of a particular metabolic pathway, functional category or operon was carried out as follows:

Let *X *denote the number of up(down)-regulated genes belonging to a metabolic pathway, cell functional category or operon. If *X *follows the commonly assumed hypergeometric distribution, then(1)

where:

*T *= total number of genes in the genome;

*M *= number of genes in the total genome known to belong to that metabolic pathways, cell functional category or operon;

*n *= total number of up(down)-regulated genes in the sample.

The probability that the number of genes associated to the node in question is equal to or greater than *k*, under the hypothesis that no differential expression took place (i.e. the *p*-value associated to an observed *k *number) can be calculated as(2)

When the *p*-value was smaller than 0.05, the *X *= *k *event being unlikely to have happened purely by chance, we considered that the metabolic pathway, cell functional category or operon was significantly differentially expressed.

### Accession Numbers

Microarray data have been deposited with Array Express [Array Express:E-MEXP-2379]

## Authors' contributions

CP conceived the study, participated in generation of experimental data, constructed and analyzed the networks and drafted the manuscript. MDR and MM-C helped to carry out experimental work. JCDH, MWP, participated in experimental design and coordination and helped to write the manuscript, NJW helped with data analysis and interpretation and manuscript writing. JB participated in conceiving the study, networks design and manuscript writing. All authors have read and approved the final manuscript.

## Supplementary Material

Additional file 1 **Fig S1: Cluster analysis**. Cluster analysis of the variation in gene transcription during the lag phase of young and old cells with respect to the stationary phase. Click here for file

Additional file 2 **Fig S2: Transcriptional network during the lag phase of young cells**. Network representation of genes (circles), operons (squares), transcription factors (octagons) and sigma factors (hexagons) at several sampling times during the lag phase of young cells. Transcription was up-regulated (green) or down-regulated (red). Arcs connect transcription factors with those genes whose transcription is initiated (green) or repressed (red) by them and sigma factors with the regulated genes (orange). The sizes of nodes are proportional to their degrees. Click here for file

Additional file 3 **Fig S3: Transcriptional network during the lag phase of old cells**. Network representation of genes (circles), operons (squares), transcription factors (octagons) and sigma factors (hexagons) at several sampling times during the lag phase of young cells. Transcription was up-regulated (green) or down-regulated (red). Arcs connect transcription factors with those genes whose transcription is initiated (green) or repressed (red) by them and sigma factors with the regulated genes (orange). The sizes of nodes are proportional to their degrees. Click here for file

Additional file 4 **Full image of Fig **2**: Gene expression during lag phase of young cells**. Symbols as in Fig 1. Symbols are coloured in green if transcription was up-regulated and in red if down-regulated. Green (red) arcs connect transcription factors with genes whose transcription is initiated (repressed). Orange arcs connect sigma factors with the regulated genes. The sizes of nodes are proportional to their degrees. Click here for file
