# Supplementary figures and images for: Network analysis of the transcriptional pattern of young and old cells of Escherichia coli during lag phase
Source: BMC Syst Biol. 2009 Nov 16;3:108. doi: 10.1186/1752-0509-3-108 (PMC2780417; doi:10.1186/1752-0509-3-108)

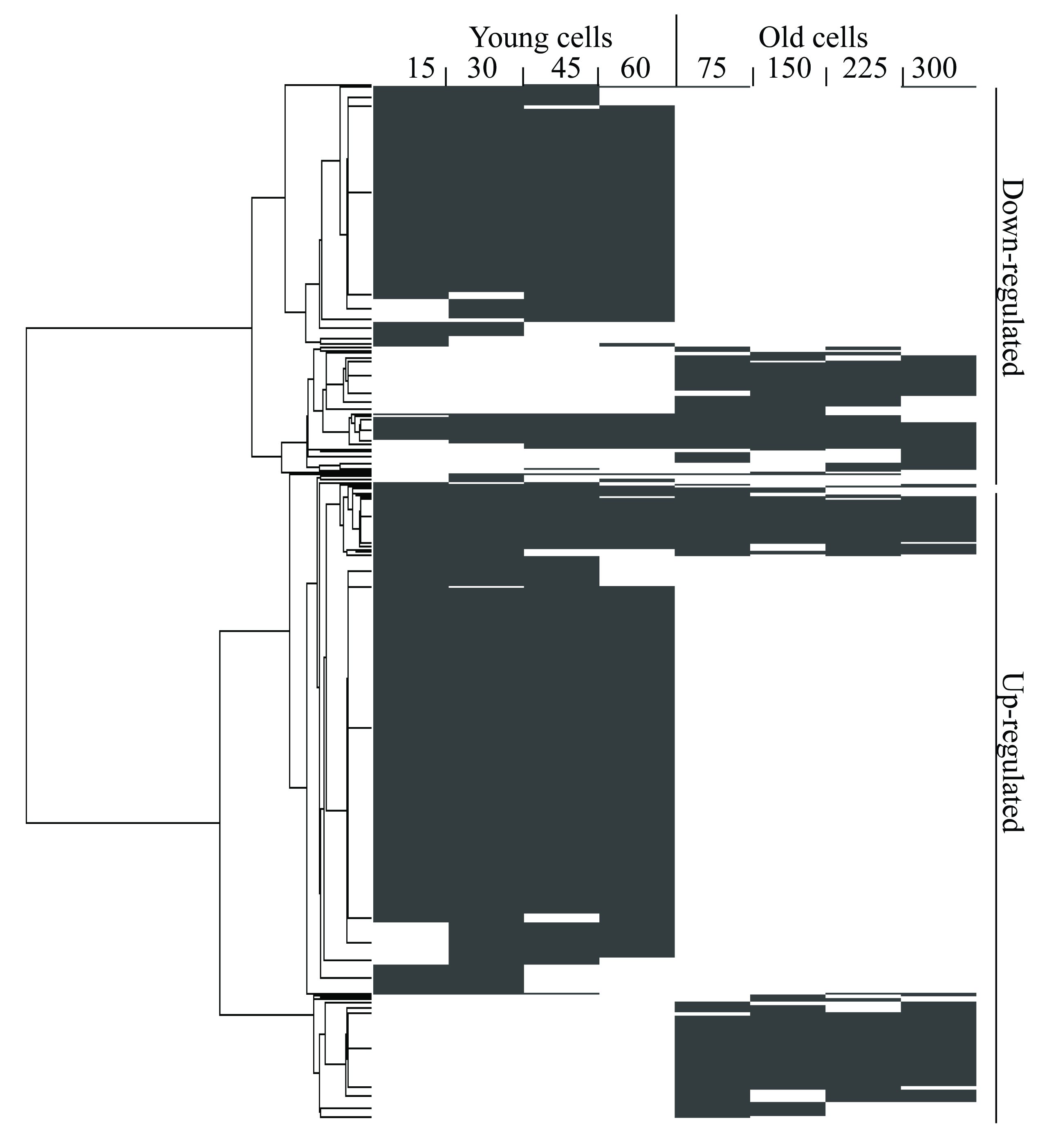

Supplement: Additional file 1 — Fig S1: Cluster analysis. Cluster analysis of the variation in gene transcription during the lag phase of young and old cells with respect to the stationary phase. [file 1752-0509-3-108-S1.JPEG]

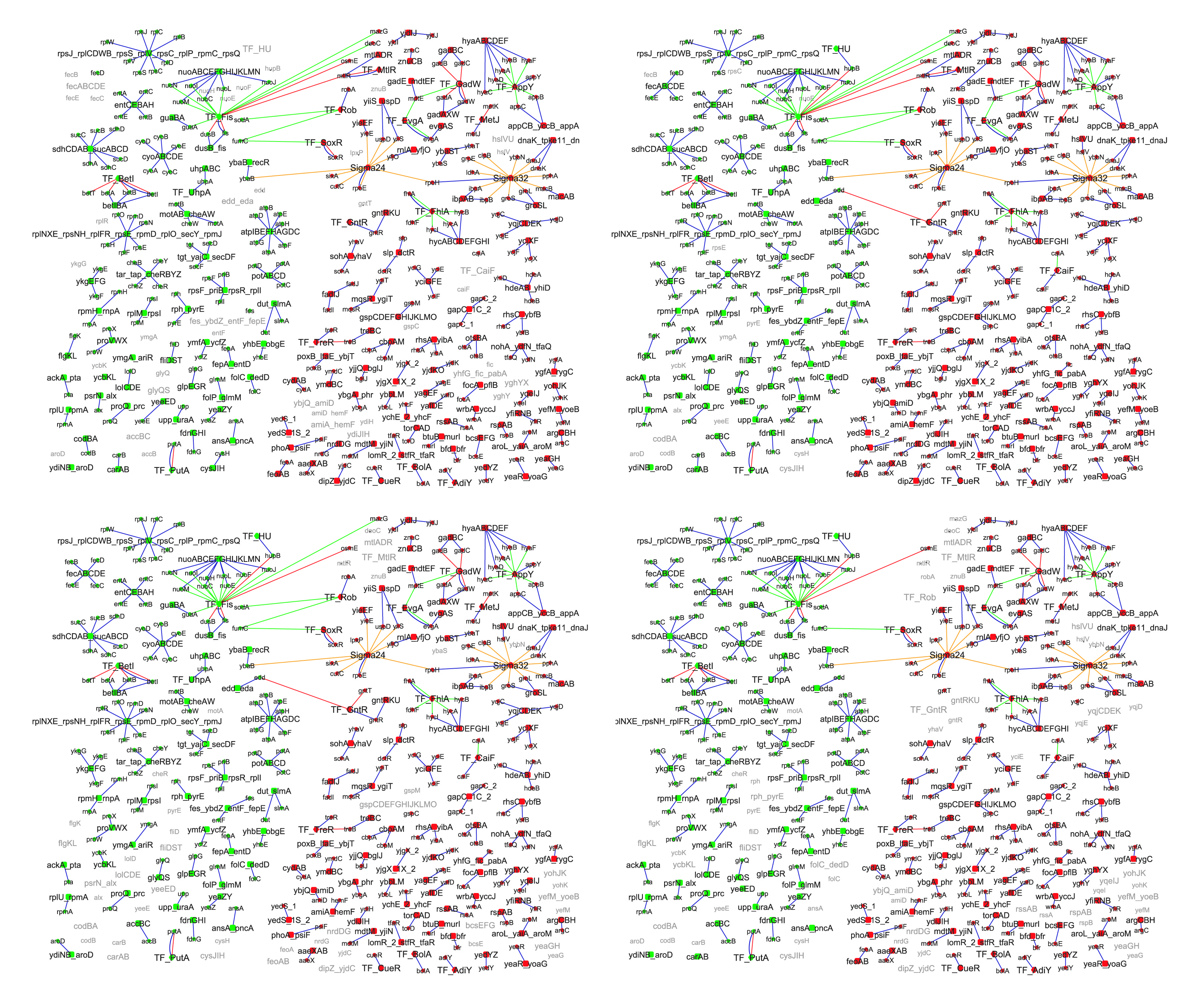

Supplement: Additional file 2 — Fig S2: Transcriptional network during the lag phase of young cells. Network representation of genes (circles), operons (squares), transcription factors (octagons) and sigma factors (hexagons) at several sampling times during the lag phase of young cells. Transcription was up-regulated (green) or down-regulated (red). Arcs connect transcription factors with those genes whose transcription is initiated (green) or repressed (red) by them and sigma factors with the regulated genes (orange). The sizes of nodes are proportional to their degrees. [file 1752-0509-3-108-S2.JPEG]

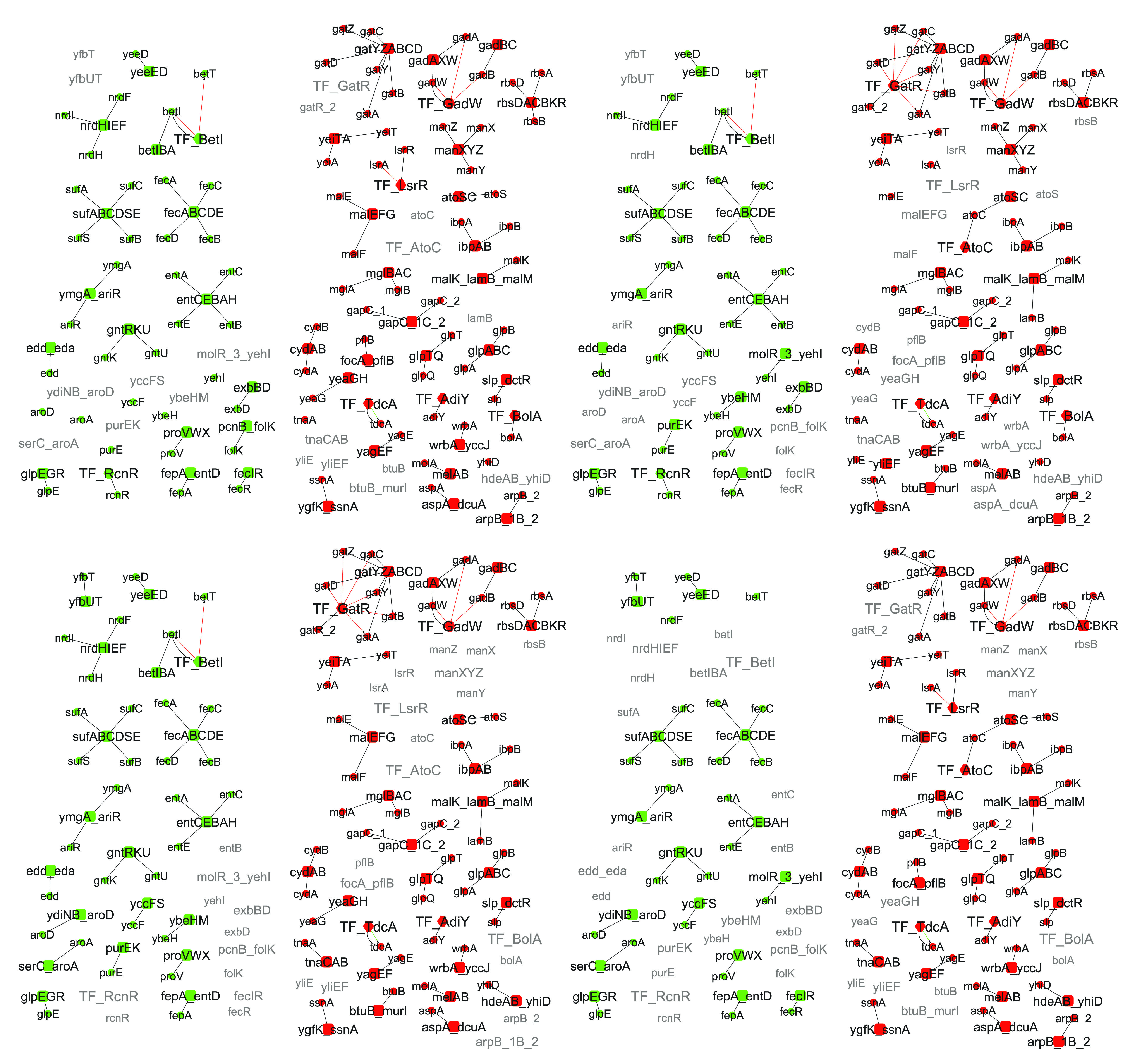

Supplement: Additional file 3 — Fig S3: Transcriptional network during the lag phase of old cells. Network representation of genes (circles), operons (squares), transcription factors (octagons) and sigma factors (hexagons) at several sampling times during the lag phase of young cells. Transcription was up-regulated (green) or down-regulated (red). Arcs connect transcription factors with those genes whose transcription is initiated (green) or repressed (red) by them and sigma factors with the regulated genes (orange). The sizes of nodes are proportional to their degrees. [file 1752-0509-3-108-S3.JPEG]

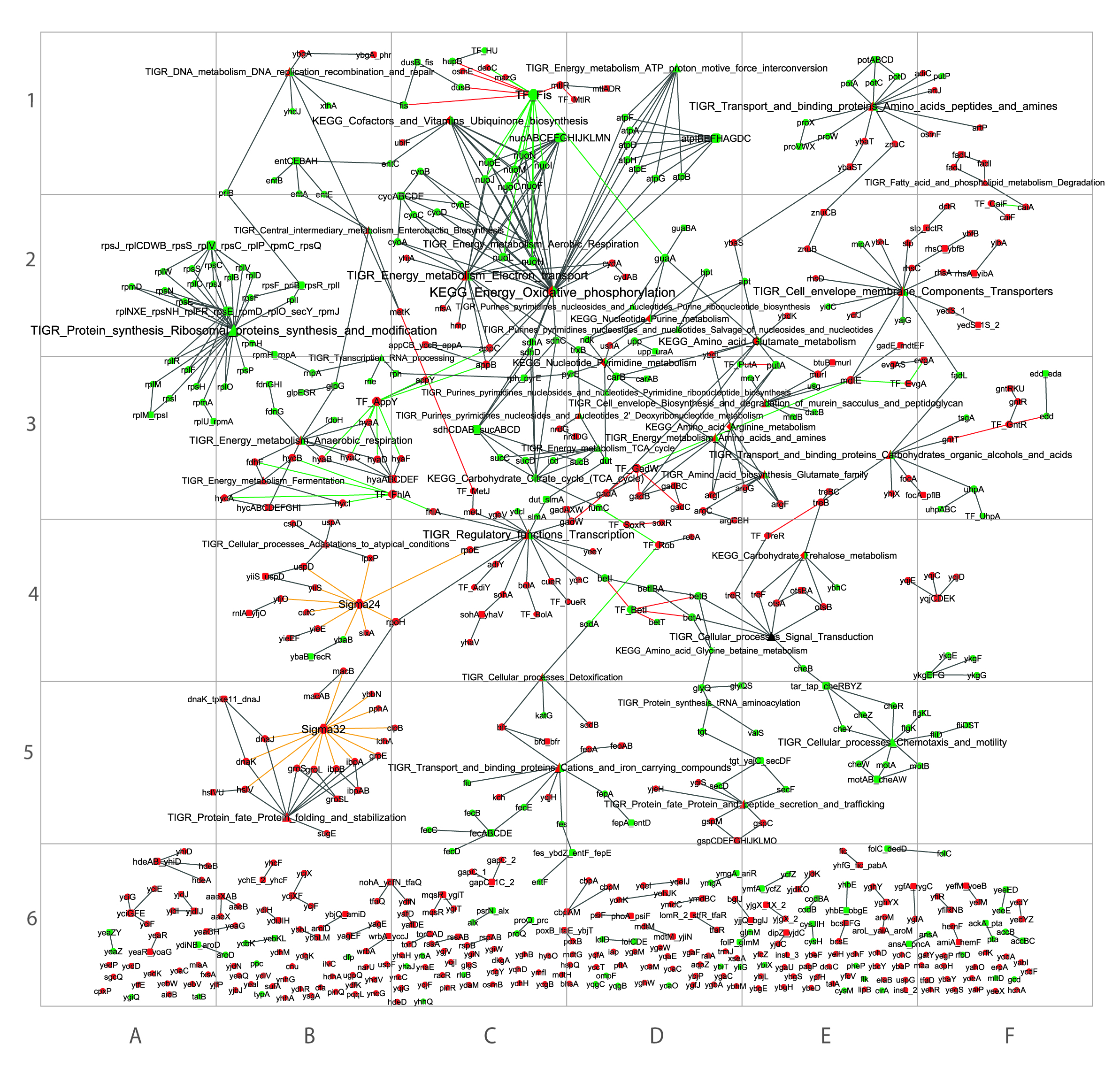

Supplement: Additional file 4 — Full image of Fig 2: Gene expression during lag phase of young cells. Symbols as in Fig 1. Symbols are coloured in green if transcription was up-regulated and in red if down-regulated. Green (red) arcs connect transcription factors with genes whose transcription is initiated (repressed). Orange arcs connect sigma factors with the regulated genes. The sizes of nodes are proportional to their degrees. [file 1752-0509-3-108-S4.JPEG]
